# Sex-Specific Association Between Socioeconomic Status, Lifestyle, and the Risk of Frailty Among the Elderly in China

**DOI:** 10.3389/fmed.2021.775518

**Published:** 2021-11-18

**Authors:** Huai-yu Wang, Mufan Zhang, Xiaojing Sun

**Affiliations:** ^1^National Institute of Health Data Science, Peking University, Beijing, China; ^2^School of Public Health, Peking University, Beijing, China; ^3^Beijing Hospital, National Center of Gerontology, Institute of Geriatric Medicine, Chinese Academy of Medical Sciences, Beijing, China; ^4^Renal Division, Department of Medicine, Peking University First Hospital, Peking University Institute of Nephrology, Beijing, China

**Keywords:** frailty, lifestyle, socioeconomic status, sex disparity, the longevous population

## Abstract

**Background:** Lifestyle contributors to frailty among the elderly were previously reported in the developed Western countries, while evidence from the less developed East Asian regions was still lacking. Due to the well-acknowledged sex-based disparity of frailty and sex-difference of socioeconomic status and lifestyle, it is worth investigating the sex-specific association between the social and behavioral contributors and the risk of frailty among the East Asian longevous population.

**Methods:** The present study was an observational study based on the four waves of interviews of the Chinese Longitudinal Healthy Longevity Survey (CLHLS) from 2008 to 2018. The participants aged ≥65 years and without frailty at baseline were included. Fried criteria (exhaustion, shrink, weakness, low mobility, and inactivity) were adopted to identify the incidence of frailty (≥3 domains) and pre-frailty (1–2 domains) during the follow-up. The sex-specific association between lifestyle (smoke status, drinking status, food intake, sleep, exercise, and physical activity) and the risk of incident pre-frailty and frailty was analyzed using the multinomial logistic regression models.

**Results:** Altogether, 3,327 participants aged 81.2 ± 10.3 (range 65–116) years were included. In total, 964 (29.0%) and 1,249 (37.5%) participants were recognized as having incident pre-frailty and frailty, respectively. Older women were disproportionately uneducated, frequently did housework and labor work, but seldom did exercise. Men had diverse dietary and recreational activities but were frequently exposed to tobacco and alcohol. The protective effects of higher income, exercise, doing housework, and daily intake of fresh fruits/vegetables were found in both the sexes (*P* < 0.05). Sleep disorders (odds ratio [*OR*] = 2.16, 95% *CI*: 1.28–3.62) and labor work (*OR* = 2.18, 95% *CI*: 1.42–3.33) were associated with the increased risk of frailty among women. For men, diverse dietary (four types of food added: *OR* = 0.21, 95% *CI*: 0.09–0.50) showed a protective effect on the risk of frailty, but daily intake of pickled vegetables showed the opposite effect (*OR* = 1.86, 95% *CI*: 1.12–3.07).

**Conclusion:** Socioeconomic status, lifestyle, and the association with the risk of frailty showed substantial difference between the sexes among the longevous population in China. To establish the individualized strategy of behavioral improvement for the frailty prevention should consider the sex disparity.

## Introduction

Frailty is an age-related syndrome characterized by the deficits of physical function, deterioration of physiological reserves, and vulnerability to stressors (1, 2). It was considered as a natural course of aging and was increasingly used as an indicator of biological aging (2, 3). Thanks to the striking development of society and medicine, global life expectancy has increased continuously (4), which is accompanied by the increase of frail population (1, 2). The prevention and intervention of frailty have become major challenges to the public health. Sex disparity of frailty and its health-mortality paradox were widely acknowledged (5, 6). In contrast to men, women were more likely to be frail but often showed the lower risk of mortality (5, 6). Although the pathophysiological pathways of frailty and the sex difference were not clear yet, the previous studies indicated the essential roles of the combination of biological, behavioral, and social factors (5).

Several clinical guidelines of frailty management suggested that the modifiable risk factors of frailty, such as unhealthy diet and insufficient physical activity, should be prevented earlier (7, 8). Multiple dietary quality scores were suggested to predict the risk of frailty (9), and an individualized physical activity program was strongly recommended to prevent and treat frailty (7). However, it should be noted that few of the previous studies included a large sample size of the longevous population (e.g., aged over 80 years) (9–14). Additionally, lifestyle is obviously affected by culture and socioeconomic status. Women, especially those residing in the less developed Asian countries, are more likely to overwork and be over-committed to family but endure the persistent shortage of care and economic support (15–18). The previous studies were mainly conducted in the developed Western countries or developed Asian countries such as Japan (9, 10, 12, 14). The sex-specific association between socioeconomic status, lifestyle and the risk of frailty among the longevous population in the less developed Asian countries is still under-investigation that means evidence for the sex-specific strategy of lifestyle improvement among the elderly in the developing countries is still lacking.

As to the largest developing country China, the Chinese population who were born in the early twentieth century and became the longevous people in the twenty-first century experienced the World War II, famine, poverty, and rapid prosperity of the country. Since the foundation of the People's Republic of China in 1949, the social status of the Chinese women has undergone enormous changes (15). Social status of women was changed from an oppressed and enslaved group in the past thousands of years to masters of their own fate. The progress of culture and socioeconomic status alters the sex-specific lifestyles and further influences the health. Insight into sex-specific socioeconomic status, lifestyle and frailty of the longevous population could not only be a supplement to the understanding of sex difference of frailty in low-income countries, but also guide the prevention and management of frailty among the East Asian older adults. The Chinese Longitudinal Healthy Longevity Survey (CLHLS) is a nationally representative cohort study in China, which recruited a large sample size of octogenarians, non-agenarians, and centenarians and followed up more than 10 years (19, 20). It provides a precious opportunity to investigate the sex-specific influence of socioeconomic status and lifestyles to the risk of frailty among the oldest-old population in a developing country. Hence, based on the CLHLS, the present study comprehensively investigated the sex-specific association among socioeconomic status, lifestyle, and the risk of frailty among the elderly in China. The effects of sex-specific dietary patterns and types of daily physical activity on the risk of frailty were further evaluated so as to provide more clues for the improvement of lifestyle.

## Methods

### Population

The present study was conducted based on the 2008–2018 cohort study of CLHLS (21). The CLHLS was conducted in a random-sample design, which recruited participants in 22 of the 31 provinces, covering about 85% population of China. Centenarians in the sampled counties and cities were invited to the survey and number-matched residents aged from 65 to 99 years living near to the centenarians were recruited either. With this design, the representativeness of the population in CLHLS was ensured. Also, social and behavioral data collected by the CLHLS are feasible to investigate the determinants of healthy aging in China (19, 21). The information of the Participants, such as demographic characteristics, socioeconomic status, lifestyle, and health status was collected using the structured questionnaires. Data from the four waves of interviews (2008, 2012, 2014, 2018) were adopted. Altogether, 9,494 participants aged ≥65 years at baseline (2008) and having complete data of frailty during the follow-up were included. The participants who had frailty (*n* = 3,647) or at the baseline of pre-frailty (*n* = 2,036) were excluded. Another 484 participants were excluded due to the absence of data related to lifestyle. Ultimately, 3,327 participants were eligible for the present study. The criteria for frailty and pre-frailty are mentioned below (Methods, *Outcome* section).

The CLHLS was approved by the Research Ethics Committee of Peking University (IRB00001052-13074) (22, 23). All the participants provided written informed consent. Detailed information of the CLHLS could be found elsewhere (20, 23).

### Covariates

The age groups were categorized into 65–79, 80–89, 90–99, and ≥100 years. Multimorbidity was defined as having more than three comorbidities, such as hypertension, diabetes, heart disease, cerebrovascular disease, chronic pulmonary disease, eye disease (cataract or glaucoma), cancer, Parkinson's disease, dementia, mental disease, arthritis, gastrointestinal ulcer, and hepatitis (24–26). Body mass index (BMI) was calculated as weight divided by height square and categorized into normal (18.5–23.9 kg/m^2^), underweight (<18.5 kg/m^2^), overweight (24.0–27.9 kg/m^2^), and obesity (≥28.0 kg/m^2^). Low accessibility of healthcare was defined if the participants answered “No” to the question “Would you timely see a doctor if you suffered from a severe illness?”

### Socioeconomic Status

The levels of education were defined as uneducated (never being educated), primary school (being educated for 1–6 years), and middle school or above (being educated for 7 years or more). Household income was recorded as quartiles.

### Lifestyles

Smoke status was recorded as never, past, and current (22). Drinking status was recorded as never, past, low risk drinking (alcohol consumption: ≤ 25 g for men, ≤ 15 g for women), and high risk drinking (alcohol consumption: >25 g for men, >15 g for women) (27). Sleep was defined as normal (5–10 h/day and no sleep disorder), excessive (>10 h/day) and insufficient (<5 h/day or having sleep disorder) (28–30). Exercise was defined as never, past, and current.

### Physical Activity

According to social and cultural background, the CLHLS collected type and frequency of the most common physical activities among the longevous population in China (21). The following physical activities were recorded in the frequency of <1 time/week or ≥1 time/week: radio/TV/reading, playing cards, social activity, outdoor activity, gardening/keeping a pet, housework/childcare, and raising domestic animals.

The intensity of activity was defined as low-to-medium intensive activity (radio/TV/reading, playing cards, social activity, outdoor activity, and gardening/keeping a pet), and high-intensive activity (housework/childcare and raising domestic animals).

### Food Intake

Food intake was recorded as daily, sometimes (weekly/monthly), or rarely intake of meat, fish/seafoods, eggs, dairy, legumes, fresh fruits/vegetables, tea, garlic, and pickled vegetables (22).

The categories of food intake were defined as the following groups: meat/fish/seafoods/eggs, fresh fruits/vegetables, diary/legumes, and tea/garlic (27). In accordance with the daily food category, dietary diversity was defined as staple food only (mainly eat rice/wheat/corn but rarely eat other types of food, such as meat, fruits/vegetables, and dairy products), one type added, two types added, three types added, and four types added. The participants added four types of food to staple food were defined as having diverse diet.

### Outcome

The modified Fried criteria (exhaustion, shrink, weakness, low mobility, and inactivity) were adopted to identify the incident pre-frailty and frailty (31).

Exhaustion was defined if the participant answered “always,” “often,” or “sometimes” to questions “I felt old and useless” or “I felt everything I did was an effort” (26, 32, 33). Shrink was defined as BMI <18.5 kg/m^2^ (26, 33). Weakness was defined if the participant was unable to lift a bag weighted 5 kg (34). Low mobility was defined if the participant was unable to walk for 1 km (35). Inactivity was defined if the participants did all the following activities less than one time/week: playing cards, social activity, gardening/keeping a pet, outdoor activity, housework/childcare, and raising domestic animals (32).

Incidence of pre-frailty and frailty was identified if the participants arose 1–2 domains and ≥3 domains during the follow-up, respectively. The participants showed no domains during the follow-up were defined as non-frailty (31, 32).

### Statistics

The demographic characteristics (age and sex), socioeconomic status (education and household income), health status (multimorbidity and accessibility of healthcare), and lifestyles (smoke status, drinking status, sleep, exercise, intensity of physical activity, dietary diversity, types of physical activity, and type of food intake) were presented according to the status of non-frailty, incident pre-frailty, and incident frailty, respectively. Chi-square tests and one-way ANOVA were applied for the comparison of categorical and normal-distributed continuous variables, respectively. The baseline characteristics were compared between the sexes (1, 5).

The multinomial logistic regression models were used to analyze the factors associated with the risk of incident pre-frailty and frailty, respectively. Following factors at baseline (2008) were analyzed as the categorical variables such as age, sex, education, household income, multimorbidity, smoke status, drinking status, sleep, exercise, intensity of physical activity, and dietary diversity. Odds ratio (*OR*) and 95% *CI* were separately calculated for the incident pre-frailty and frailty, each in reference to non-frailty. The sex-stratified analyses were conducted.

The association among the types of physical activity, types of food intake at baseline (2008), and the risk of incident pre-frailty and frailty was analyzed using the multinomial regression models, respectively. The covariates, such as age, sex, education, household income, and multimorbidity were adjusted. The results were presented in *OR* and 95% *CI*. The sex-stratified analyses were conducted.

All the analyses were two tailed and *p* value < 0.05 was considered to be statistically significant. All the statistical analyses were performed using Stata version 16.0 (Stata Corp LP, College Station, TX, USA).

## Results

### Population Characteristics

In total, 3,327 participants with an age of 81.2 ± 10.3 (range 65–116) years were included. Among them, 1,011 (30.4%) were aged 90–99 years and 321 (9.7%) were centenarians (Table 1). Till 2018, 964 (29.0%) and 1,249 (37.5%) participants were recognized as incident pre-frailty and incident frailty, respectively. Compared with the non-frail participants, those with incident pre-frailty were significantly younger, whereas those with incident frailty were significantly older (*P* < 0.001) (Table 1). Men (57.2%) and women (61.5%) were predominant among pre-frail and frail population, respectively (*P* < 0.001) (Table 1).

**Table 1:** Comparison of the characteristics at baseline according to the status of frailty during the follow-up.

**Characteristics**	**Overall**	**Non-frailty**	**Pre-frailty**	**Frailty**	***P* value**
In total (*n*, %)	3,327 (100.0)	1,114 (33.5)	964 (29.0)	1,249 (37.5)	
Mean age (years, mean ± SD)	81.2 ± 10.3	81.3 ± 10.9	75.8 ± 8.7	85.4 ± 8.9	<0.001
**Age group (** ***n*** **, %)**					<0.001
65–79 years	1,060 (31.9)	375 (33.7)	525 (54.5)	160 (12.8)	
80–89 years	935 (28.1)	268 (24.1)	272 (28.2)	395 (31.6)	
90–99 years	1,011 (30.4)	356 (32.0)	139 (14.4)	516 (41.3)	
≥100 years	321 (9.7)	115 (10.3)	28 (2.9)	178 (14.3)	
**Sex (** ***n*** **, %)**					<0.001
Men	1,684 (50.6)	649 (58.3)	551 (57.2)	484 (38.8)	
Women	1,643 (49.4)	465 (41.7)	413 (42.8)	765 (61.3)	
Multimorbidity (*n*, %)	664 (21.1)	220 (20.7)	178 (19.3)	266 (22.8)	0.141
Uneducated (*n*, %)	1,600 (35.5)	462 (41.5)	348 (36.1)	790 (63.3)	<0.001
Low household income (*n*, %)^†^	858 (25.8)	208 (18.7)	251 (26.0)	399 (32.0)	<0.001
Low accessibility to healthcare (*n*, %)	115 (3.5)	21 (1.9)	18 (1.9)	76 (6.1)	<0.001
Having diverse diet (*n*, %)^‡^	400 (12.0)	174 (15.6)	128 (13.3)	98 (7.9)	<0.001
**Smoke status (** ***n*** **, %)**					<0.001
Never	2,052 (61.7)	672 (60.3)	533 (55.3)	847 (67.8)	
Past	566 (17.0)	213 (19.1)	157 (16.3)	196 (15.7)	
Current	709 (21.3)	229 (20.6)	274 (28.4)	206 (16.5)	
**Drinking status (n, %)**					<0.001
Never	2,188 (66.9)	714 (65.1)	572 (60.9)	902 (73.0)	
Past	432 (13.2)	155 (14.1)	122 (13.0)	155 (12.6)	
Low risk drinking	159 (4.9)	68 (6.2)	48 (5.1)	43 (3.5)	
High risk drinking	492 (15.0)	160 (14.6)	197 (21.0)	135 (10.9)	
High-intensive activity (n, %)	2,519 (77.6)	842 (75.6)	818 (84.9)	859 (83.4)	<0.001
**Exercise (n, %)**					<0.001
Never	1,805 (54.3)	504 (45.2)	523 (54.3)	778 (62.3)	
Past	331 (10.0)	97 (8.7)	78 (8.1)	156 (12.5)	
Current	1,191 (35.8)	513 (46.1)	363 (37.7)	315 (25.2)	
**Sleep disorder (n, %)**					<0.001
Excessiveness	187 (5.6)	65 (5.8)	47 (4.9)	75 (6.0)	
Insufficiency	320 (9.6)	72 (6.5)	82 (8.5)	166 (13.3)	

*†Low household income: the participants whose household income <25% of the population were defined as having low household income.*

*‡Diverse diet: based on staple food, the participants added four types of food were defined as having diverse diet*.

The highest proportions of current smokers (28.4%) and excessive drinkers (21.0%) were observed among the population with incident pre-frailty. Compared with the non-frail participants, those with incident frailty were more likely to be inactive, have single diet and insufficient sleep (*P* < 0.001) (Table 1). The proportions of participants raising domestic animals daily were higher in the groups of incident pre-frailty (33.7%) and incident frailty (26.8%) as compared with the non-frail group (22.9%) (*P* < 0.001) (Table 2).

**Table 2:** Comparison of types of food intake and types of physical activity at baseline according to the status of frailty during the follow-up.

**Types (*n*, %)**	**Overall**	**Non-frailty**	**Pre-frailty**	**Frailty**	***p* value**
**Physical activity**					
**Radio/TV/reading**					<0.001
<1 time/week	757 (22.8)	191 (17.2)	127 (13.2)	439 (35.2)	
≥1 times/week	2,570 (77.3)	923 (82.9)	837 (86.8)	810 (64.9)	
**Playing cards**					<0.001
<1 time/week	2,824 (84.9)	907 (81.4)	787 (81.6)	1,130 (90.5)	
≥1 times/week	503 (15.1)	207 (18.6)	177 (18.4)	119 (9.5)	
**Social activity**					<0.001
<1 time/week	3,042 (91.4)	977 (87.7)	861 (89.3)	1,204 (96.4)	
≥1 times/week	285 (8.6)	137 (12.3)	103 (10.7)	45 (3.6)	
**Gardening/keeping a pet**					<0.001
<1 time/week	2,738 (82.3)	851 (76.4)	758 (78.6)	1,129 (90.4)	
≥1 times/week	589 (17.7)	263 (23.6)	206 (21.4)	120 (9.6)	
**Outdoor activity**					<0.001
<1 time/week	1,022 (30.7)	262 (23.5)	287 (29.8)	473 (37.9)	
≥1 times/week	2,305 (69.3)	852 (76.5)	677 (70.2)	776 (62.1)	
**Housework/childcare**					<0.001
<1 time/week	951 (28.6)	313 (28.1)	184 (19.1)	454 (36.4)	
≥1 times/week	2,376 (71.4)	801 (71.9)	780 (80.9)	795 (63.7)	
**Raising domestic animals**					<0.001
<1 time/week	2,412 (72.5)	859 (77.1)	639 (66.3)	914 (73.2)	
≥1 times/week	915 (27.5)	255 (22.9)	325 (33.7)	335 (26.8)	
**Food intake**					
**Meat**					<0.001
Rarely	604 (18.2)	178 (16.0)	143 (14.8)	283 (22.7)	
Sometimes	1,656 (49.8)	539 (48.4)	501 (52.0)	616 (49.3)	
Daily	1,067 (32.1)	397 (35.6)	320 (33.2)	350 (28.0)	
**Fish/seafoods**					<0.001
Rarely	1,148 (34.5)	341 (30.6)	296 (30.7)	511 (40.9)	
Sometimes	1,894 (56.9)	651 (58.4)	583 (60.5)	660 (52.8)	
Daily	285 (8.6)	122 (11.0)	85 (8.8)	78 (6.2)	
**Eggs**					0.071
Rarely	576 (17.3)	189 (17.0)	147 (15.3)	240 (19.2)	
Sometimes	1,558 (46.8)	508 (45.6)	460 (47.7)	590 (47.2)	
Daily	1,193 (35.9)	417 (37.4)	357 (37.0)	419 (33.6)	
**Dairy**					<0.001
Rarely	2,044 (61.4)	628 (56.4)	587 (60.9)	829 (66.4)	
Sometimes	589 (17.7)	178 (16.0)	193 (20.0)	218 (17.5)	
Daily	694 (20.9)	308 (27.7)	184 (19.1)	202 (16.2)	
**Legumes**					0.025
Rarely	898 (27.0)	301 (27.0)	231 (24.0)	366 (29.3)	
Sometimes	1,760 (52.9)	572 (51.4)	532 (55.2)	656 (52.5)	
Daily	669 (20.1)	241 (21.6)	201 (20.9)	227 (18.2)	
**Fresh fruits/vegetables**					<0.001
Rarely	668 (20.1)	161 (14.5)	171 (17.7)	336 (26.9)	
Sometimes	2,100 (63.1)	707 (63.5)	621 (64.4)	772 (61.8)	
Daily	559 (16.8)	246 (22.1)	172 (17.8)	141 (11.3)	
**Tea**					<0.001
Rarely	1,736 (52.2)	551 (49.5)	433 (44.9)	752 (60.2)	
Sometimes	281 (8.5)	91 (8.2)	92 (9.5)	98 (7.9)	
Daily	1,310 (39.4)	472 (42.4)	439 (45.5)	399 (32)	
**Garlic**					<0.001
Rarely	1,440 (43.3)	453 (40.7)	375 (38.9)	612 (49)	
Sometimes	1,110 (33.4)	371 (33.3)	362 (37.6)	377 (30.2)	
Daily	777 (23.4)	290 (26)	227 (23.6)	260 (20.8)	
**Pickled vegetables**					<0.001
Rarely	1,764 (53.0)	649 (58.3)	449 (46.6)	666 (53.3)	
Sometimes	898 (27.0)	283 (25.4)	283 (29.4)	332 (26.6)	
Daily	665 (20.0)	182 (16.3)	232 (24.1)	251 (20.1)	

### Sex-Specific Characteristics at Baseline

**Figure 1:**
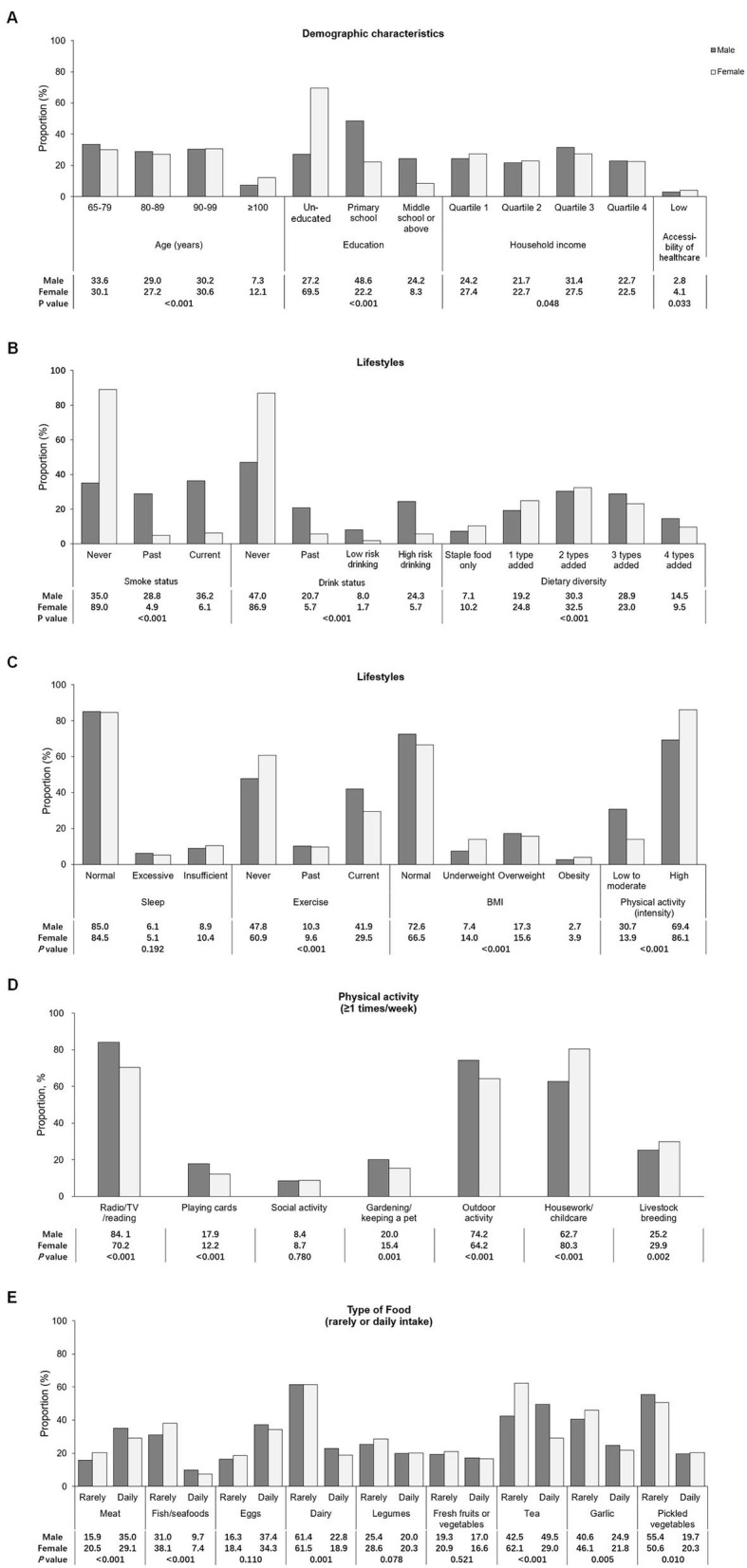
Comparison of characteristics between genders. **(A)** The demographic characteristics and accessibility of healthcare. **(B)** Lifestyles, such as the status of smoke, drinking, and the diversity of diet. **(C)** Lifestyles, such as sleep conditions, habits of exercise, and daily physical activity categorized by intensity and the category of body mass index. **(D)** Type of daily physical activity. **(E)** Type of food intake. The comparison of types of food intake between genders was conducted using three groups of intake including rarely, sometimes and daily, of which the significance was presented in the current figure. The percentage of rarely and daily intake was presented using the bars whereas the percentage of food sometimes intake was not shown in this figure.

Comparison of characteristics between sexes is shown in Figure 1. Compared with men, more women were centenarians (12.1 vs. 7.3%), uneducated (69.5 vs. 27.2%), and more likely to have low accessibility of healthcare (4.1 vs. 2.8%) (Figure 1A).

Men were more frequently exposed to tobacco and alcohol but had a diversified diet (*P* < 0.001) (Figure 1B). Women were more likely to have insufficient sleep, did high-intensive activity but never did exercise (*P* < 0.001) (Figure 1C). Men preferred sedentary or recreational activities, whereas women frequently did high-intensive activities such as housework/childcare and raising domestic animals (*P* < 0.001) (Figure 1D). Men consumed more nutritious food, whereas women mostly had pickled vegetables (*P* < 0.001) (Figure 1E).

### Socioeconomic Status

As to the risk of incident pre-frailty, those aged over 80 years and with higher levels of household income were associated with the reduced risk of incident pre-frailty (*P* < 0.05) (Table 3).

**Table 3:** Factors associated with pre-frailty, frailty, and sex stratified.

**Variables**	**Overall**				**Men**				**Women**			
	**Pre-frailty** **vs. non-frailty**		**Frailty** **vs. non-frailty**		**Pre-frailty** **vs. non-frailty**		**Frailty** **vs. non-frailty**		**Pre-frailty** **vs. non-frailty**		**Frailty** **vs. non-frailty**	
	**OR (95% CI)**	***P* value**	**OR (95% CI)**	***P* value**	**OR (95% CI)**	***P* value**	**OR (95% CI)**	***P* value**	**OR (95% CI)**	***P* value**	**OR (95% CI)**	***P* value**
**Socioeconomic status**												
**Education**												
Uneducated	Ref.	Ref.	Ref.	Ref.	Ref.	Ref.	Ref.	Ref.	Ref.	Ref.	Ref.	Ref.
Primary school	1.26 (0.90–1.76)	0.178	0.89 (0.66–1.21)	0.455	1.64 (1.02–2.64)	0.043	1.18 (0.77–1.81)	0.452	1.00 (0.60–1.65)	0.992	0.70 (0.44–1.09)	0.114
Middle school or above	1.11 (0.74–1.68)	0.611	0.53 (0.35–0.81)	0.004	1.54 (0.87–2.70)	0.136	0.68 (0.38–1.19)	0.175	0.79 (0.41–1.54)	0.486	0.39 (0.19–0.77)	0.007
**Household income**												
Quartile 1	Ref.	Ref.	Ref.	Ref.	Ref.	Ref.	Ref.	Ref.	Ref.	Ref.	Ref.	Ref.
Quartile 2	0.66 (0.44–1.00)	0.052	0.61 (0.41–0.89)	0.011	0.67 (0.38–1.19)	0.171	0.55 (0.31–0.97)	0.037	0.69 (0.36–1.31)	0.256	0.68 (0.40–1.17)	0.165
Quartile 3	0.49 (0.33–0.73)	<0.001	0.57 (0.40–0.81)	0.002	0.42 (0.24–0.71)	0.001	0.59 (0.35–0.98)	0.043	0.57 (0.32–1.04)	0.065	0.59 (0.36–0.98)	0.041
Quartile 4	0.59 (0.39–0.89)	0.013	0.45 (0.31–0.66)	<0.001	0.49 (0.27–0.86)	0.014	0.39 (0.22–0.70)	0.002	0.68 (0.37–1.25)	0.218	0.52 (0.31–0.87)	0.012
**Lifestyles**												
**Smoke status**												
Never	Ref.	Ref.	Ref.	Ref.	Ref.	Ref.	Ref.	Ref.	Ref.	Ref.	Ref.	Ref.
Past	1.04 (0.69–1.58)	0.843	1.33 (0.89–1.98)	0.166	1.30 (0.81–2.08)	0.276	1.42 (0.88–2.28)	0.151	0.52 (0.17–1.56)	0.242	1.33 (0.57–3.12)	0.515
Current	1.24 (0.84–1.83)	0.283	1.34 (0.90–1.98)	0.146	1.54 (0.97–2.43)	0.065	1.67 (1.04–2.68)	0.034	1.22 (0.55–2.69)	0.625	0.73 (0.33–1.61)	0.438
**Drinking**												
Never	Ref.	Ref.	Ref.	Ref.	Ref.	Ref.	Ref.	Ref.	Ref.	Ref.	Ref.	Ref.
Past	0.89 (0.58–1.37)	0.59	0.86 (0.57–1.29)	0.456	0.80 (0.49–1.33)	0.394	1.03 (0.64–1.68)	0.896	1.17 (0.49–2.79)	0.721	0.52 (0.23–1.19)	0.121
Low risk drinking	1.13 (0.61–2.10)	0.701	0.88 (0.49–1.58)	0.657	1.01 (0.51–1.99)	0.977	0.88 (0.44–1.77)	0.724	1.39 (0.26–7.35)	0.696	1.13 (0.32–3.94)	0.853
High risk drinking	1.84 (1.24–2.75)	0.003	0.81 (0.53–1.24)	0.335	1.88 (1.18–2.99)	0.008	1.02 (0.62–1.69)	0.941	1.46 (0.63–3.37)	0.381	0.41 (0.18–0.91)	0.030
**Sleep**												
Normal	Ref.	Ref.	Ref.	Ref.	Ref.	Ref.	Ref.	Ref.	Ref.	Ref.	Ref.	Ref.
Excessive	0.97 (0.52–1.81)	0.918	0.67 (0.39–1.17)	0.163	0.90 (0.42–1.96)	0.799	0.51 (0.22–1.19)	0.119	1.10 (0.37–3.23)	0.867	0.79 (0.36–1.74)	0.560
Insufficient	1.27 (0.84–1.92)	0.263	1.75 (1.20–2.54)	0.003	1.47 (0.82–2.63)	0.195	1.42 (0.81–2.51)	0.224	1.07 (0.58–1.98)	0.833	2.16 (1.28–3.62)	0.004
**Exercise**												
Never	Ref.	Ref.	Ref.	Ref.	Ref.	Ref.	Ref.	Ref.	Ref.	Ref.	Ref.	Ref.
Past	0.85 (0.52–1.40)	0.528	1.36 (0.89–2.06)	0.153	0.70 (0.35–1.43)	0.333	1.46 (0.81–2.64)	0.206	1.09 (0.53–2.25)	0.822	1.30 (0.70–2.41)	0.402
Current	0.97 (0.72–1.31)	0.855	0.60 (0.45–0.79)	<0.001	1.21 (0.81–1.81)	0.361	0.66 (0.44–0.99)	0.047	0.81 (0.51–1.28)	0.359	0.59 (0.40–0.88)	0.009
**Physical activity**												
Low to medium	Ref.	Ref.	Ref.	Ref.	Ref.	Ref.	Ref.	Ref.	Ref.	Ref.	Ref.	Ref.
High	0.99 (0.69–1.42)	0.936	0.61 (0.45–0.83)	0.002	0.99 (0.65–1.51)	0.949	0.67 (0.45–1.00)	0.049	0.86 (0.39–1.9)	0.706	0.46 (0.27–0.77)	0.003
**Dietary diversity**												
Staple food only	Ref.	Ref.	Ref.	Ref.	Ref.	Ref.	Ref.	Ref.	Ref.	Ref.	Ref.	Ref.
1 type added	0.92 (0.50–1.68)	0.781	0.74 (0.44–1.22)	0.235	1.21 (0.49–3.00)	0.684	0.53 (0.24–1.18)	0.121	0.65 (0.28–1.51)	0.319	0.89 (0.46–1.69)	0.716
2 types added	1.04 (0.59–1.86)	0.887	0.59 (0.36–0.96)	0.034	0.77 (0.32–1.85)	0.555	0.37 (0.18–0.80)	0.011	1.43 (0.65–3.17)	0.377	0.75 (0.40–1.41)	0.364
3 types added	1.26 (0.70–2.27)	0.436	0.58 (0.35–0.95)	0.031	0.79 (0.33–1.92)	0.605	0.33 (0.15–0.71)	0.005	2.11 (0.93–4.80)	0.075	0.87 (0.44–1.69)	0.672
4 types added	0.93 (0.48–1.79)	0.830	0.41 (0.23–0.75)	0.003	0.69 (0.27–1.76)	0.436	0.21 (0.09–0.50)	<0.001	1.42 (0.52–3.85)	0.493	0.78 (0.32–1.92)	0.590

*Adjusted for age, sex, and multimorbidity*.

As to the risk of incident frailty, women sex and aging were the risk predictors (*P* < 0.001). High levels of education and household income were associated with the reduced risk of frailty (*P* < 0.05). The sex-stratified analyses showed that the levels of education were more influential to women, whereas the levels of income affected men more (*P* < 0.001) (Table 3).

### Lifestyles

High risk drinking was associated with the increased risk of incident pre-frailty (*OR* = 1.84, 95% *CI*: 1.24–2.75). Current smoke was associated with the increased risk of frailty among men (*OR* = 1.67, 95% *CI*: 1.04–2.68). Insufficient sleep was the risk factor of frailty (*OR* = 1.75, 95% *CI*: 1.20–2.54), especially among women (*OR* = 2.16, 95% *CI*: 1.28–3.62). Currently, doing sport (*OR* = 0.60, 95% *CI*: 0.45–0.79) and high-intensive activity (*OR* = 0.61, 95% *CI*: 0.45–0.83) showed strongly protective effects on frailty. Diversified diet was associated with the reduced risk of frailty among men (four types added: *OR* = 0.21, 95% *CI*: 0.09–0.50), but not for women (*P* > 0.05) (Table 3).

### Physical Activity

As to the risk of incident pre-frailty, social activity showed the protective effect (*OR* = 0.72, 95% *CI*: 0.52–0.98) while raising domestic animals it showed an adverse effect (*OR* = 1.41, 95% *CI*: 1.03–1.93) (Table 4).

**Table 4:** Association among the types of physical activity, types of food intake and the risk of pre-frailty, frailty, and sex stratified.

**Variables**	**Overall**				**Men**				**Women**			
	**Pre-frailty** **vs. non-frailty**		**Frailty** **vs. non-frailty**		**Pre-frailty** **vs. non-frailty**		**Frailty** **vs. non–frailty**		**Pre–frailty** **vs. non–frailty**		**Frailty** **vs. non–frailty**	
	**OR (95% CI)**	***P* value**	**OR (95% CI)**	***P* value**	**OR (95% CI)**	***P* value**	**OR (95% CI)**	***P* value**	**OR (95% CI)**	***P* value**	**OR (95% CI)**	***P* value**
**Types of physical activity (Ref**. ** <1 times/week)**												
Radio/TV/reading	1.37 (0.90–2.08)	0.142	0.67 (0.48–0.92)	0.013	0.97 (0.52–1.82)	0.929	0.48 (0.29–0.82)	0.007	1.71 (0.97–3.04)	0.066	0.82 (0.54–1.24)	0.340
Playing cards	1.03 (0.74–1.45)	0.857	0.77 (0.54–1.10)	0.156	1.13 (0.73–1.74)	0.586	0.85 (0.51–1.41)	0.522	0.90 (0.52–1.57)	0.716	0.71 (0.42–1.20)	0.200
Social activity	0.91 (0.61–1.36)	0.646	0.71 (0.45–1.12)	0.141	1.22 (0.71–2.09)	0.474	0.57 (0.26–1.27)	0.170	0.70 (0.38–1.29)	0.254	0.72 (0.40–1.30)	0.275
Gardening/keeping a pet	1.04 (0.77–1.41)	0.793	0.51 (0.36–0.71)	<0.001	1.01 (0.68–1.51)	0.948	0.32 (0.19–0.54)	<0.001	1.10 (0.67–1.81)	0.715	0.79 (0.49–1.29)	0.351
Outdoor activity	0.72 (0.52–0.98)	0.039	0.54 (0.40–0.71)	<0.001	0.79 (0.50–1.25)	0.310	0.45 (0.29–0.70)	<0.001	0.66 (0.42–1.05)	0.077	0.64 (0.43–0.95)	0.027
Housework/childcare	0.87 (0.63–1.21)	0.415	0.49 (0.36–0.65)	<0.001	0.95 (0.65–1.38)	0.772	0.60 (0.41–0.88)	0.009	0.70 (0.35–1.41)	0.316	0.36 (0.22–0.58)	<0.001
Raising domestic animals	1.41 (1.03–1.93)	0.031	1.69 (1.25–2.29)	0.001	1.02 (0.66–1.56)	0.944	1.24 (0.78–1.96)	0.368	2.01 (1.25–3.23)	0.004	2.18 (1.42–3.33)	<0.001
**Food intake (Ref. rarely)**											
**Meat**												
Sometimes	1.28 (0.86–1.91)	0.221	0.79 (0.56–1.13)	0.194	1.15 (0.66–1.99)	0.631	0.70 (0.42–1.19)	0.189	1.55 (0.85–2.83)	0.151	0.89 (0.54–1.47)	0.660
Daily	1.23 (0.79–1.90)	0.357	0.90 (0.61–1.32)	0.590	1.12 (0.61–2.05)	0.708	0.86 (0.49–1.52)	0.610	1.43 (0.74–2.77)	0.290	0.97 (0.57–1.68)	0.927
**Fish/seafoods**												
Sometimes	0.91 (0.65–1.27)	0.591	1.20 (0.89–1.61)	0.243	1.27 (0.80–1.99)	0.311	1.38 (0.89–2.15)	0.150	0.60 (0.36–1.01)	0.053	1.02 (0.67–1.56)	0.915
Daily	0.76 (0.45–1.26)	0.283	0.77 (0.47–1.27)	0.307	0.70 (0.36–1.38)	0.308	0.60 (0.28–1.28)	0.184	0.80 (0.35–1.84)	0.595	0.93 (0.45–1.92)	0.842
**Eggs**												
Sometimes	1.02 (0.68–1.53)	0.925	1.02 (0.71–1.48)	0.903	1.01 (0.58–1.76)	0.965	1.06 (0.61–1.84)	0.837	1.02 (0.55–1.87)	0.960	1.02 (0.61–1.69)	0.948
Daily	1.16 (0.76–1.77)	0.488	1.07 (0.73–1.57)	0.725	0.98 (0.55–1.74)	0.936	0.84 (0.47–1.49)	0.549	1.44 (0.76–2.75)	0.263	1.45 (0.85–2.45)	0.170
**Dairy**												
Sometimes	0.86 (0.58–1.27)	0.455	0.79 (0.55–1.13)	0.200	1.05 (0.61–1.81)	0.855	0.94 (0.54–1.61)	0.808	0.85 (0.47–1.52)	0.586	0.69 (0.42–1.12)	0.132
Daily	0.74 (0.52–1.05)	0.093	0.53 (0.38–0.74)	<0.001	0.66 (0.41–1.05)	0.080	0.45 (0.28–0.73)	0.001	0.81 (0.46–1.42)	0.455	0.64 (0.39–1.03)	0.068
**Legumes**												
Sometimes	1.43 (1.02–2.02)	0.038	1.56 (1.15–2.12)	0.004	1.3 (0.82–2.06)	0.268	1.80 (1.14–2.85)	0.012	1.63 (0.96–2.74)	0.069	1.35 (0.88–2.07)	0.172
Daily	1.10 (0.72–1.70)	0.653	1.32 (0.89–1.95)	0.170	1.02 (0.57–1.84)	0.948	1.33 (0.74–2.41)	0.341	1.2 (0.62–2.32)	0.599	1.28 (0.73–2.23)	0.385
**Fresh fruits/vegetables**												
Sometimes	0.97 (0.67–1.39)	0.849	0.58 (0.42–0.79)	0.001	0.70 (0.44–1.13)	0.148	0.5 (0.32–0.79)	0.003	1.36 (0.76–2.45)	0.298	0.63 (0.41–0.98)	0.040
Daily	1.05 (0.66–1.67)	0.843	0.43 (0.28–0.67)	<0.001	0.69 (0.37–1.30)	0.249	0.51 (0.27–0.95)	0.034	1.76 (0.85–3.67)	0.128	0.39 (0.21–0.73)	0.004
**Tea**												
Sometimes	1.48 (0.91–2.41)	0.117	0.90 (0.55–1.46)	0.658	1.47 (0.74–2.93)	0.268	0.95 (0.46–1.96)	0.884	1.53 (0.74–3.16)	0.246	0.87 (0.44–1.71)	0.692
Daily	1.3 (0.97–1.74)	0.080	0.98 (0.74–1.29)	0.882	1.06 (0.72–1.56)	0.757	0.89 (0.61–1.30)	0.555	1.92 (1.19–3.08)	0.007	1.21 (0.79–1.85)	0.383
**Garlic**												
Sometimes	0.94 (0.69–1.29)	0.710	0.88 (0.65–1.18)	0.388	0.88 (0.58–1.36)	0.573	0.83 (0.54–1.28)	0.400	0.96 (0.59–1.56)	0.870	0.87 (0.57–1.32)	0.509
Daily	0.74 (0.52–1.03)	0.077	0.79 (0.58–1.09)	0.154	0.64 (0.40–1.01)	0.057	0.65 (0.41–1.05)	0.078	0.88 (0.52–1.49)	0.622	0.94 (0.6–1.48)	0.800
**Pickled vegetables**												
Sometimes	1.16 (0.83–1.61)	0.386	0.96 (0.71–1.31)	0.812	1.36 (0.87–2.12)	0.177	1.04 (0.66–1.64)	0.850	0.92 (0.55–1.52)	0.740	0.91 (0.59–1.41)	0.683
Daily	1.69 (1.19–2.41)	0.004	1.38 (0.98–1.95)	0.064	2.15 (1.33–3.48)	0.002	1.86 (1.12–3.07)	0.016	1.22 (0.71–2.11)	0.476	1.06 (0.65–1.72)	0.813

*Adjusted for age, sex, education, household income, and multimorbidity*.

As to the risk of incident frailty, physical activities including radio/TV/reading, gardening/keeping a pet, outdoor activity and housework/childcare were significantly associated with the reduced risk of frailty (*P* < 0.001). Benefits of radio/TV/reading and gardening/keeping a pet on the incident frailty were observed among men (*P* < 0.001), but not among women. Raising domestic animals was significantly associated with the increased risk of frailty (*OR* = 1.69, 95% *CI*: 1.25–2.29), especially among women (*OR* = 2.18, 95% *CI*: 1.42–3.33) (Table 4).

### Food Intake

Daily intake of pickled vegetables was associated with the increased risk of incident pre-frailty (*OR* = 1.69, 95% *CI*: 1.19–2.41), especially among men (*OR* = 2.15, 95% *CI*: 1.33–3.48) (Table 4).

Sometimes or daily intake of fresh fruits/vegetables showed the protective effect on the incident frailty in both sexes (*P* < 0.05). Daily intake of dairy was associated with the reduced risk of frailty among men (*OR* = 0.45, 95% *CI*: 0.28–0.73), while daily intake of pickled vegetables showed adverse effect on the risk of frailty among men (*P* < 0.05) (Table 4).

## Discussion

Based on the nationally representative cohort of the elderly in China, the present study comprehensively investigated the sex-specific association between socioeconomic status, lifestyle, and the risk of pre-frailty and frailty, respectively. The protective effects of high levels of income, exercise, high-intensive activity, and fresh fruits/vegetables on the risk of frailty were found in both the sexes. For older women, the improvement in education and sleep and the avoidance of labor work might be beneficial for the prevention of frailty. For older men, cessation of tobacco, reduction of pickled vegetables' intake but increase of dairy intake might be beneficial for the prevention of frailty.

The disparity of frailty between sexes was well-recognized (2, 5, 36, 37). The present results of sex disparity are consistent with the previous studies, while its underlying reasons seem different from developed countries. Various studies in Western countries reported that much positive healthcare-seeking behavior and better perception of healthcare partly contributed to the high rate of diagnosis and early intervention of frailty among women (5). This phenomenon suggested the influence of awareness and the use of healthcare resources on the management of frailty (5, 38). However, in the present study, the Chinese older women were disproportionately uneducated, and more women showed low accessibility of healthcare as compared with men, especially among rural residents. The proportion of uneducated women in rural areas was 62.7% while in urban areas it was 37.7%. A higher proportion of women residents in rural areas (5.75%) showed low accessibility of healthcare compared with that in urban areas (2.17%). Besides, proportions of un-education among women having low income in urban and rural areas were 81.5 and 80.6%, respectively. Hence, the high rate of incident frailty among women in the present study was more likely to be the integrated consequence of the long-term exposure to a series of unhealthy lifestyles and limited resources. According to the present results, women frequently did high-intensive activities including housework, childcare, and raising domestic animal but infrequently did exercise, which was more likely to accumulate the functional and physical impairments and further led to the high incidence of frailty. Generally, higher income was considered as the protective factor of frailty (2, 39), while in the present study, women were less influenced by household income compared with men. It might be a consequence of the women adaptability, generated from the long-term enduring of limited economic and care resources. It should be noted that the studied population was born from the 1890s to the 1940s. In that era, the turmoil of Chinese society strongly limited the education and economy. Additionally, the lagging feudal thought of “men are priority to women” exacerbated the problem of un-education and shortage of resources among women. It should be noted that the enormous improvement of sex disparity of socioeconomic status has been achieved during the past 70 years (15, 40). The association between socioeconomic status and the risk of frailty and its underlying reasons in the present results might be different among the population who were born in the new era of China (e.g., people born in the 1990s experienced flourishing of China after the reform and opening-up policy).

The significant differences in lifestyle and their association with frailty were found between sexes among older adults in China. In contrast to men, less diverse diet and less intake of nutritious food, such as meat, fish/seafoods, dairy products, tea, and garlic, were found among older women. Age-related reduction of appetite might contribute to the less diverse diet among older women (7). Besides, the determinants of socioeconomic factors on eating habits should be considered. Although women had significantly less diverse diet, the milder influence of dietary diversity on the risk of frailty was found among women. Meanwhile, the less influence of income on the risk of frailty further evidenced adaptability of women to behavioral nutrition based on a relatively poor socioeconomic status. The highest percentage of current smokers, mainly men, was found in the population with incident pre-frailty. Cessation of tobacco use among the elderly should be further intensified.

By analyzing the patterns of physical activity, the present study found that the men pattern of activity was much similar to the sedentary lifestyle, whereas women did more high-intensive activities including housework/childcare and raising domestic animals. Compared with men, fewer older women spent time on reading, watching TV, or listening to the radio. The levels of education may contribute to the low utilization of media among women, and the beneficial effects of media among men might be resourced from the healthcare information acquired from these media. It was noteworthy that women did more high-intensive activities compared with men, but only a few of them did exercise. Additionally, the risk effect of labor work was found among older women. Given the higher rate of sarcopenia among older women in contrast to men (5), it raised the concern that whether the excessive activity based on the relatively insufficient physiological reserve played adverse roles in the pathogenesis of frailty. Nascimento et al. reported the importance of exercise on the intervention of both sarcopenia and frailty (41). Combined with the current results, we recommended the avoidance of labor work and the increase of exercise among older women.

Collectively, the present study revealed the sex-specific evidence for the improvement of diet and physical activity among the elderly in China, which would supplement the existing policy and guidelines for healthy aging. The Chinese government previously released a detailed plan called “Healthy China Initiative (2019–2030),” which presented the recommendations on diet, physical activity, social support, and healthcare for healthy aging in detail (42, 43). Intake of meat, seafood, egg, milk, and legume was recommended by the Initiative. According to the present results, intake of fresh fruit and vegetables should be also recommended and the use of pickled vegetables should be reduced. As to physical activity, in addition to exercise and physical training recommended by the Initiative, benefits of activity in daily life, such as housework, gardening, and outdoor activity, were observed in the present study, which should be supplemented in the recommendations. Considering the lower socioeconomic levels of aging women in China compared with men, forces from the society, community, and family should take part in the support of aging women to modify their lifestyles and prevention from chronic diseases including frailty.

It has to be admitted that the present study has limitations. First, only self-reported frequency of food intake was recorded. Data of processing and cooking method and quantity of intake, which may alter the nutrients of the identical food, were unavailable in the present study. Lack of detailed data might be the reason because no significant association between the daily intake of meat and reduced risk of frailty was found in the present study. Second, data on diet and physical activity were collected by questionnaires, recall bias exists. Third, the data of outcomes (incident pre-frailty and incident frailty) were collected from the interviews of 2011, 2014, and 2018. The exact dates of outcomes were unavailable. Hence, the logistic regression models, instead of Cox regression models, were adopted in the present study. Fourth, multimorbidity was defined using self-reported data and its prevalence might be underestimated because of awareness. In a previous study, Herr et al. reported that around 50% of the population aged over 70 years had at least three comorbidities (26), while the prevalence of multimorbidity in the present study was 21.1%. The underestimated prevalence might be influenced the results on the association between multimorbidity and the risk of frailty. Lastly, subject to the observational feature, no causal conclusion could be made in the present study.

## Conclusion

In conclusion, socioeconomic status and lifestyle were significantly associated with the incidence of frailty among the elderly in China. Social and behavioral factors which should be improved varied between genders. Individualized strategy for the frailty prevention should consider the substantial sex disparity of socioeconomic status, lifestyle, and its association with frailty.

## Data Availability Statement

Publicly available datasets were analyzed in this study. This data can be found at: https://opendata.pku.edu.cn/dataverse/CHADS.

## Ethics Statement

The studies involving human participants were reviewed and approved by Research Ethics Committee of Peking University. The patients/participants provided their written informed consent to participate in this study.

## Author Contributions

HYW designed the study, analyzed the data, interpreted the results, wrote the manuscript, and is study guarantor. MZ and XS interpreted the results and revised the manuscript. All authors contributed to the article and approved the submitted version.

## Funding

This study was supported by the National Natural Science Foundation of China (82100741 and 82000668).

## Conflict of Interest

The authors declare that the research was conducted in the absence of any commercial or financial relationships that could be construed as a potential conflict of interest.

## Publisher's Note

All claims expressed in this article are solely those of the authors and do not necessarily represent those of their affiliated organizations, or those of the publisher, the editors and the reviewers. Any product that may be evaluated in this article, or claim that may be made by its manufacturer, is not guaranteed or endorsed by the publisher.
